# Combined lymphocyte/monocyte count, D-dimer and iron status predict COVID-19 course and outcome in a long-term care facility

**DOI:** 10.1186/s12967-021-02744-2

**Published:** 2021-02-17

**Authors:** Flavia Biamonte, Cirino Botta, Maria Mazzitelli, Salvatore Rotundo, Enrico Maria Trecarichi, Daniela Foti, Carlo Torti, Giuseppe Viglietto, Daniele Torella, Francesco Costanzo

**Affiliations:** 1 Department of Experimental and Clinical Medicine, Magna Graecia University, Catanzaro, Italy; 2 Annunziata Hospital, Cosenza, Italy; 3 Unit of Infectious and Tropical Diseases, Department of Medical and Surgical Sciences, Magna Graecia University, Catanzaro, Italy; 4 Department of Health Sciences, Magna Græcia University of Catanzaro, Catanzaro, Italy; 5 Center of Interdepartmental Services (CIS), “Magna Graecia” University of Catanzaro, Catanzaro, Italy

**Keywords:** COVID-19, Clinical outcome, Long-term care facilities, Iron, Lymphocyte, Monocyte, D-dimer

## Abstract

**Background:**

The Sars-CoV-2 can cause severe pneumonia with multiorgan disease; thus, the identification of clinical and laboratory predictors of the progression towards severe and fatal forms of this illness is needed. Here, we retrospectively evaluated and integrated laboratory parameters of 45 elderly subjects from a long-term care facility with Sars-CoV-2 outbreak and spread, to identify potential common patterns of systemic response able to better stratify patients’ clinical course and outcome.

**Methods:**

Baseline white blood cells, granulocytes’, lymphocytes’, and platelets’ counts, hemoglobin, total iron, ferritin, D-dimer, and interleukin-6 concentration were used to generate a principal component analysis. Statistical analysis was performed by using R statistical package version 4.0.

**Results:**

We identified 3 laboratory patterns of response, renamed as low-risk, intermediate-risk, and high-risk, strongly associated with patients’ survival (p < 0.01). D-dimer, iron status, lymphocyte/monocyte count represented the main markers discriminating high- and low-risk groups. Patients belonging to the high-risk group presented a significantly longer time to ferritin decrease (p: 0.047). Iron-to-ferritin-ratio (IFR) significantly segregated recovered and dead patients in the intermediate-risk group (p: 0.012).

**Conclusions:**

Our data suggest that a combination of few laboratory parameters, i.e. iron status, D-dimer and lymphocyte/monocyte count at admission and during the hospital stay, can predict clinical progression in COVID-19.

## Background

Severe acute respiratory syndrome coronavirus 2 (Sars-CoV-2), the viral agent causing the novel coronavirus disease 2019 (COVID-19), has generated an ongoing worldwide pandemic after its initial outbreak in Wuhan, China [1, 2].

Diagnosis of COVID-19 is typically performed using polymerase chain reaction testing via nasopharyngeal swab. However, because of the false-negative test result rates of Sars-CoV-2 PCR testing by nasopharyngeal swabs, several clinical, laboratory, and imaging findings have been useful to make a presumptive diagnosis [3, 4]. Sars-CoV-2 infection may be asymptomatic, or it may cause a wide spectrum of symptoms, such as fever, dry cough, and shortness of breath. COVID-19 severity can also progress to severe and eventually critical conditions defined by respiratory failure, septic shock, and/or multiple organ dysfunction [5–9]. It is therefore of paramount importance for clinicians to establish reliable predictors of the progression of this illness for timely clinical/therapeutic decision-making. Common laboratory abnormalities among hospitalized patients include lymphopenia (83%), elevated inflammatory markers (eg., erythrocyte sedimentation rate, C-reactive protein, ferritin, tumor necrosis factor-α, IL-1, IL-6), and abnormal coagulation parameters (eg., prolonged prothrombin time, thrombocytopenia, elevated D-dimer, low fibrinogen). Most of these laboratory characteristics are nonspecific and are common in pneumonia. Aside from the established clinical risk factors, lymphopenia (absolute lymphocyte count < 1.0 × 10^9^/L) is the sole cardinal laboratory finding with prognostic potential [1, 10–13]. Furthermore, different acute phase biomarkers, like ferritin, and a hypercoagulability state, indicated by elevated D-dimer, have all been associated with greater illness severity and mortality [12, 13].

In the present study, we retrospectively evaluated and integrated laboratory parameters/variables of 45 COVID-19 patients admitted to our hospital, consisting of a homogenous group of elderly subjects from a long-term care facility with Sars-CoV-2 outbreak and spread, to identify potential common patterns of systemic response able to better stratify patients’ clinical course and outcome, independently from clinical co-morbidities.

## Methods

All methods were carried out in accordance with relevant guidelines and regulations.

### Patients

This study was carried out at the ‘COVID unit’ of the Magna Graecia University—“Mater Domini” Hospital in Catanzaro, Italy from March 9th to April 1st, 2020. The study was approved by the Magna Graecia University institutional ethics committee, and a waiver of informed consent was obtained by the committee.

All patients included in this case series had a Sars-Cov-2 laboratory-confirmed infection diagnosed by quantitative reverse transcriptase-PCR (qRT-PCR) (GeneFinderTM COVID-19 Plus RealAmp Kit, Elitech Group) performed on nasopharyngeal specimens or bronchoalveolar lavage (BAL).

### Clinical and laboratory assessment

Basic information such as gender, age, clinical symptoms, and signs was collected from the admission records. Admission testing included in the integrated analysis were hematologic parameters (i.e. white blood count (WBC), granulocyte count, lymphocyte count, monocyte count, platelets count, neutrophil-to-lymphocyte ratio (NLR), monocyte-to-lymphocyte ratio (MLR) platelet-to-lymphocyte ratio (PLR), hemoglobin (HGB), and iron) and inflammatory markers (i.e. ferritin, IL-6, and D-dimer) which were repeated along with the hospitalization.

### Statistical analysis

Statistical analysis was performed by using R statistical package version 4.0 [14]. Specifically, laboratory data known to be associated with inflammation and routinely performed in COVID-19 patients (white blood cells, neutrophils, lymphocytes, platelets counts, hemoglobin, ferritin, iron, D-dimer, and IL-6) were scaled to be used for PCA estimation. On these results, patients were clustered into 3 groups according to the K-means algorithm. Next, Student’s t-test and ANOVA (according to the number of groups to be tested) were used to evaluate differences in the means of the different variables. Pearson’s Chi-Square was used for comparisons between categorical variables. Correlation between variables was assessed by Pearson’s correlation test. Kaplan–Meier estimate and logrank tests were used for time to events (ferritin, D-dimer, and IL-6 decrease) calculation. The following packages have been used during the whole analysis: base package to perform Student’s t-test, ANOVA and Pearson’s tests; ggpubr and ggplot2, for figure preparation; corrplot and corrr for correlation analysis; survminer for Kaplan Meier and logrank tests; cluster and ggfortify for PCA calculation and K-means based clustering, ggradar for radar plot.

## Results

### Patients’ characteristics at baseline

Fifty patients were admitted to our institution following a COVID-19 outbreak that occurred in a long-term care facility. Of them, 45 patients with positive PCR Sars-Cov-2 laboratory-confirmed infection, presented all the required laboratory variables at baseline and further presented D-dimer, IL-6, and ferritin during the follow-up; thus, they were included in this retrospective analysis. Complete clinical data together with relative therapy have been recently published by Trecarichi et al. [15]. Patients’ characteristics at baseline are reported in Table 1.

**Table 1:** Demographic and clinical patients’ characteristics at baseline

	Patients (n = 45)
Age, years (median and range)	81 (55–98)
Sex	
Male	19 (42%)
Female	26 (58%)
Comorbidities	
Hypertension	33 (73%)
Type 2 DM	9 (20%)
Malignancy	7 (16%)
COPD	7 (16%)
CKD	19 (42%)
Obesity	5 (11%)
Neurological dis	23 (51%)
Psychiatric dis	11 (24%)
Outcome	
Recovered	33 (73%)
Deaths	12 (27%)

*Type 2 DM* Type 2 Diabetes Mellitus, *COPD* Chronic Obstructive Pulmonary Disease, *CKD* chronic kidney disease

Briefly, 19 males and 26 females with a median age of 81 years (range 55–98 years) and a median time from symptoms appearance to admission to hospital of 5 days were included in our analysis. Among them, 33 (73%) were under treatment for hypertension, 23 (51%) presented neurological diseases, 19 (43%) had a chronic kidney failure and 11 (24%) assumed drugs for psychiatric disorders. The overall mortality rate was 26.67%, in line with that reported according to the median age of Italy [16].

### Laboratory pattern of response to Sars-CoV-2 infection

**Fig. 1:**
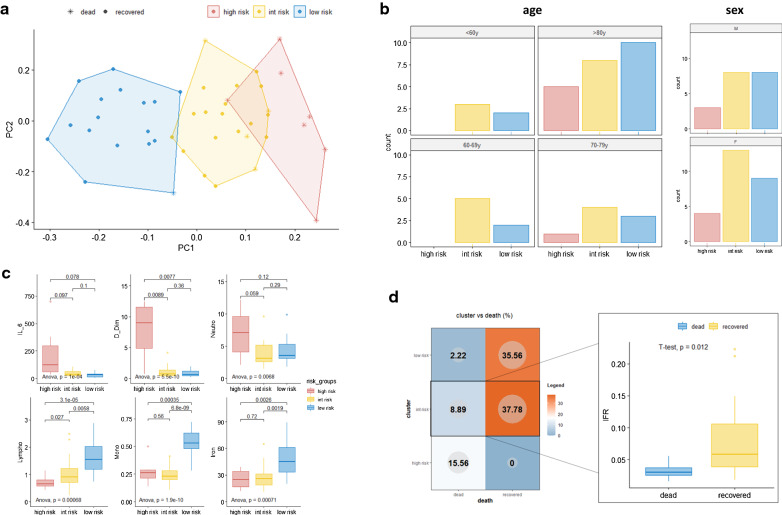
Baseline laboratory parameters define different patterns of response to Sars-CoV-2 infection. **a** Clustering analysis by a principal component analysis (PCA) scatter plot. Colours and shapes respectively represent the 3 risk groups and the status of the patient (recovered or dead). **b** distribution of patients according to risk group and age or sex. **c** ANOVA and T-test results of the 6 parameters which mainly influenced patients’ clustering, according to their distribution across risk groups. Within each sub-panel, p values for ANOVA comparison (bottom) and for each pairwise comparison (top) are reported. **d** Patient distribution according to risk groups and dead/recovered status; on the right side, the histogram shows the comparison of iron to ferritin ratio (IFR) between recovered and dead patients within the intermediate-risk group

We used baseline values of blood cell count and several biochemical and coagulation-related parameters, widely used for COVID-19 patient assessment to investigate the occurrence of a specific pattern of response. To this aim, we took advantage of a dimensionality reduction approach, the PCA, which by segregating each patient according to their whole laboratory profile (the more similar are the profiles, the closer they are on the graph) allowed us to identify potential subgroups (which could be subsequently identified through k-means approach). Specifically, white blood cells, granulocytes, lymphocytes, and platelets count, hemoglobin, total iron, ferritin, D-dimer, and interleukin 6 concentration were used to automatically generate a PCA and to cluster patients into 3 different and well-separated groups (Fig. 1a). The 3 clusters were found to be strongly associated with patients’ survival (Chi-squared test p < 0.001) and were thus renamed as low risk (1 death over 17 patients), intermediate-risk (4 deaths over 21 patients) and high risk (7 deaths over 7 patients) (patients distribution according to age and sex are reported in Fig. 1b). As shown in Fig. 1c, 6 out of 9 variables significantly discriminated the 3 groups, with D-dimer, lymphocytes/monocytes count, and iron status representing the main markers of the high- and low-risk group, respectively. Within the intermediate-risk group we registered 4 deaths, which led us to hypothesize that the clustering algorithm presents a reduced sensitivity for “borderline” patients. Indeed, we assume that that in this group the presence of a “smoldering” systemic inflammation could have been hidden by other confounding factors such as iron deficiency anemia, which by reducing the values of serum ferritin impairs its potential use as inflammatory marker, or iron supplementation, which could lead to the opposite phenomenon. To this end, we evaluated the difference in iron to ferritin ratio (IFR) as a surrogate marker of inflammation. Indeed, the increase of ferritin uncoupled from an iron increase (or in the presence of low iron values) leads to low IFR values and is usually associated with chronic inflammation [15, 16]. Of note, we observed that the difference in IFR significantly segregates recovered and dead patients (high IFR better prognosis) in the intermediate-risk group (p: 0.012) (Fig. 1d).

### Time to ferritin decrease correlates with patients’ survival

**Fig. 2:**
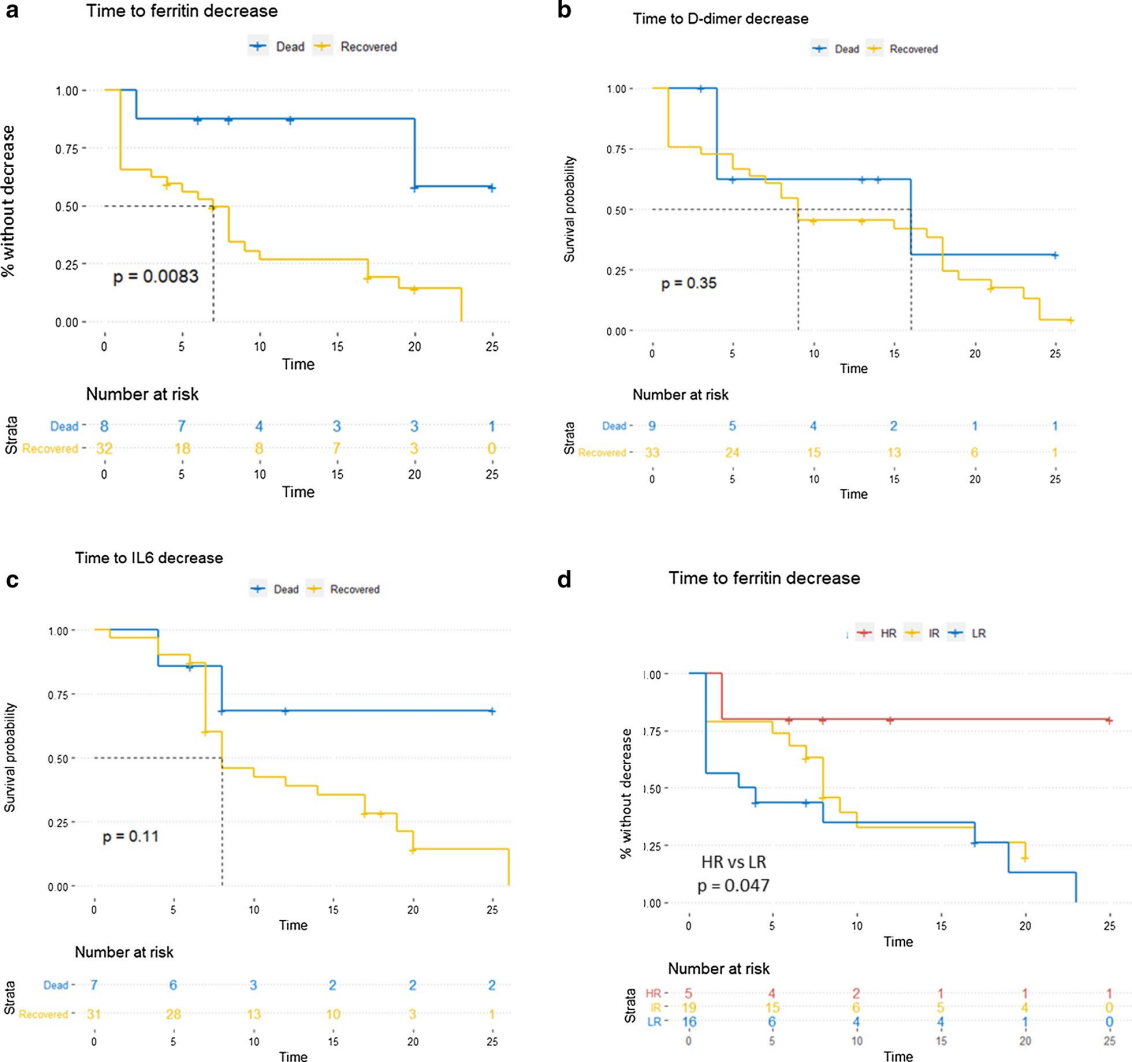
Patients with a worse outcome present a significantly longer time to ferritin decrease. **a**–**c** Kaplan Meier curves reporting the time to ferritin, D-dimer and IL-6 decrease (intended as the time from hospital admission to first and stable decrease during hospitalization) respectively, stratified on dead/recovered status (p value calculated through LogRank test). **d** Kaplan Meier curves reporting the time to ferritin decrease stratified on risk groups (p value calculated through LogRank test comparing High-Risk vs. Low-Risk patients)

In the attempt to identify informative markers useful for monitoring illness severity and mortality, we investigated the modulation of ferritin serum concentration during hospitalization. Interestingly, we observed a quick ferritin increase followed by a *decalage* phase almost exclusively in recovering patients, which indeed presented a significantly short time to ferritin decrease (intended as the time from hospital admission to first and stable ferritin decrease during hospitalization) (Fig. 2a). This trend was not observed neither for D-dimer nor for IL-6 concentration along hospitalization (Fig. 2b, c). Accordingly, patients belonging to the high-risk group presented a significantly longer time to ferritin decrease (Fig. 2d, p: 0.047).

### Correlation analyses identify lympho/monocytes count, D-dimer, and iron status as “litmus paper” of the systemic response to SARS-CoV-2 infection

**Fig. 3:**
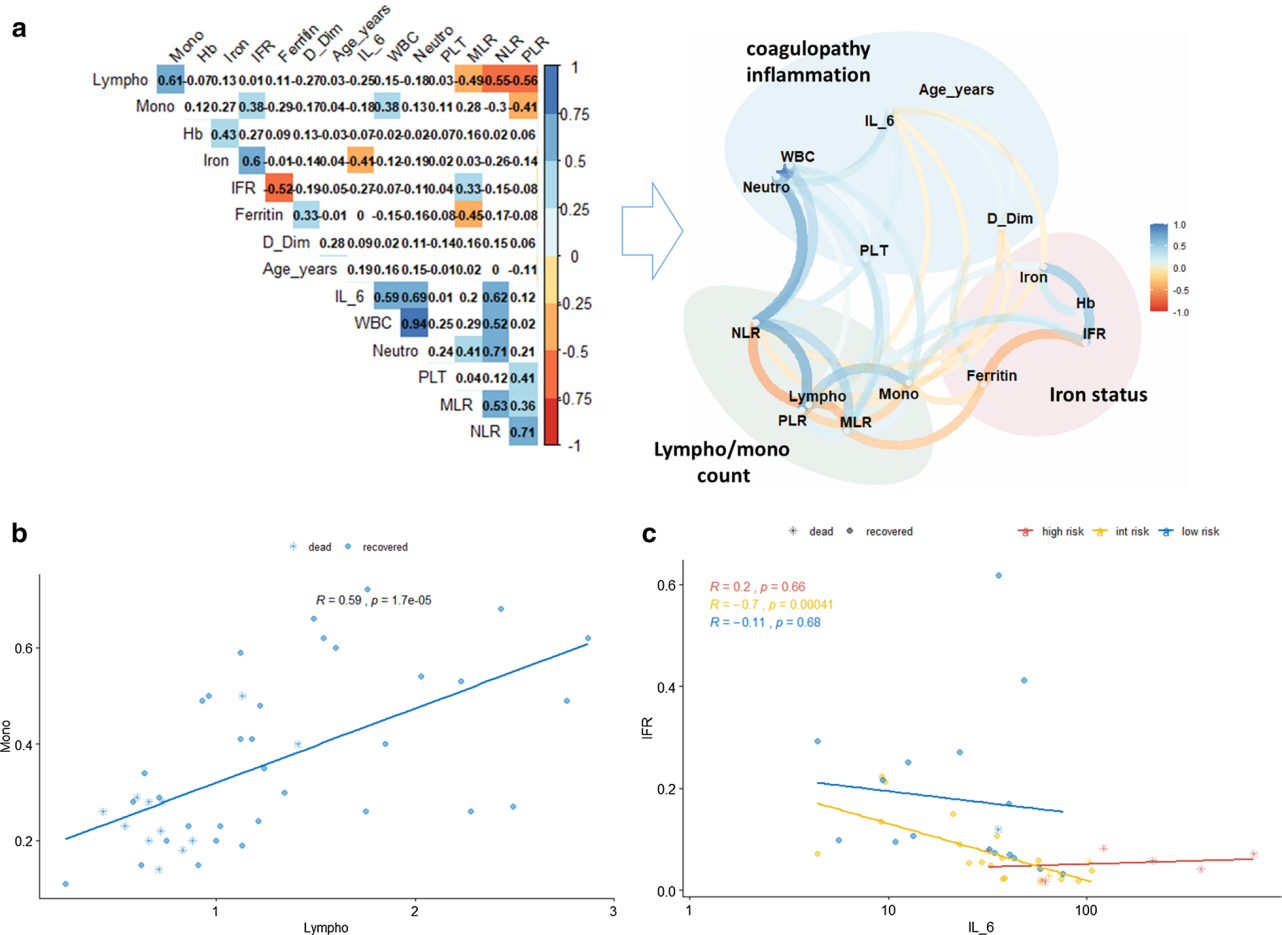
Correlations among lymph/monocytes count, D-dimer, and iron status affect the systemic response to SARS-CoV-2 infection. **a** Left: correlation plot of all variables [including derivative variables such as iron to ferritin ratio (IFR), neutrophils to lymphocytes ratio (NLR), platelets to lymphocytes ratio (PLR), monocytes to lymphocytes ratio (MLR)] to identify potential inter-variables correlations. The colour represents the direction of the correlation. Only boxes showing a significant correlation have been coloured; right, network plot showing direction and distribution of correlations between variables. Clusters of variables belonging to similar functions have been grouped. **b** Scatter plot representing the correlation between lymphocytes and monocytes. Remarkably, almost all the deaths are localized within the lower left quadrant of the plot. **c** Scatter plot showing the association between IL-6 and IFR. This correlation reaches statistical significance within the intermediate group only, further supporting the discriminating role of IFR within this subgroup

Lastly, we performed a correlation analysis, including derivative variables known to be associated with systemic inflammation, such as neutrophils, monocytes, and platelets to lymphocytes ratio (NLR, MLR, and PLR respectively). Interestingly, as shown in Fig. 3a, we observed the presence of three different clusters of correlated variables, which have been grouped accordingly to the main systemic function/role they are involved in. Among the different correlations observed, lymphocytes and monocytes experienced a similar modulation, possibly dependent on the mechanism of action of the virus. This strong relationship has been observed independently of the risk group (Fig. 3b). Moreover, we observed a direct correlation between IFR and IL-6 only in the intermediate risk group, further underscoring the role of IFR in discriminating “hidden” inflammatory responses (Fig. 3c). Other potential associations between laboratory variables and Sex, Age, risk groups, and recovery/death have been explored and reported in Additional file 1: Figures S1–S4. Briefly, we observed, among others, a direct correlation between the amount of IL-6 and D-dimer which is statistically significant in female patients only, a strong association between monocyte and lymphocyte counts (which is independent from all other confounding variables and that could represent a sign of mononucleated cells extravasation), an inverse correlation between IFR and ferritin and a direct correlation between neutrophil count and IL-6 concentration in patients who recovered from lung disease (which could represent a normal control and functionality of the iron and IL-6 homeostasis).

Altogether, these results further underscore the relevant role of these markers in identifying critical patients who potentially could benefit from increased monitoring and early intervention.

### Comorbidities are associated with the presence of markers of the high-risk group at baseline

**Fig. 4:**
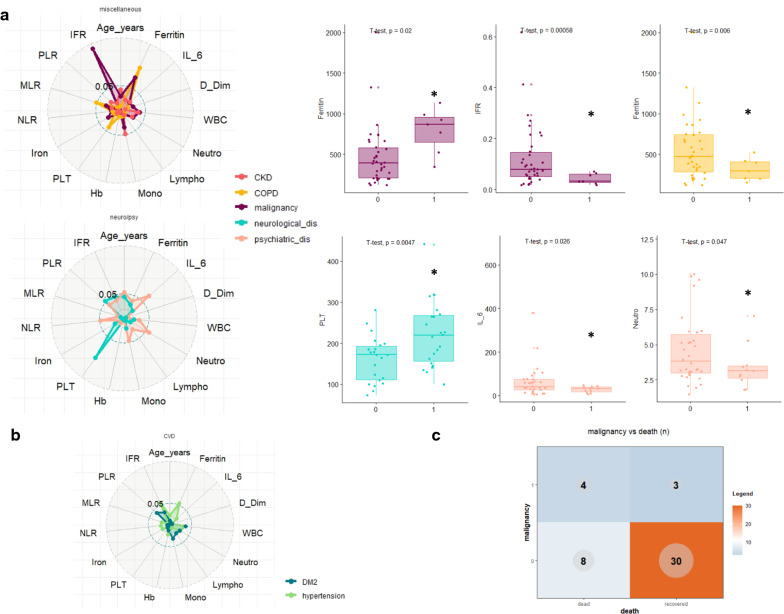
Association between comorbidities and baseline levels of laboratory variables. **a** Distribution of different laboratory values according to baseline comorbidities: left, radar-plot showing the p values of the t-test comparing each laboratory variable with each comorbidity. The distance from the center correlates with the significance of the test (the more is far the more is significant); right, box plot representing the distribution of only the significant variables in patients presenting with (1) or without (0) comorbidities. **b** Radar-plot reporting CVD (cardiovascular disease) associated variables: none of them presents a significantly different distribution of laboratory variables. **c** Distribution of COVID-19-associated deaths according to the presence or absence of malignancies at baseline

To investigate if the presence of previous comorbidities is associated with changes in baseline levels of inflammatory variables (thus potentially affecting patients’ outcome), we performed a multiple t-test, evaluating every single variable for the association to each condition.Results are reported in Fig. 4a, b as radarplot evaluating the impact of neurological and/or psychiatric disorders, cardiovascular-metabolic disease and of a miscellaneous group which includes malignancies, chronic lung disease and chronic kidney disease. Among others, the presence of a concomitant malignancy is associated with high levels of ferritin and low levels of IFR, and, in line with other reports [17] correlates with a worse survival (Fig. 4c, Additional file 1: Table S1, p: 0.047). Interestingly, we observed that the presence of hypertension correlates with a worse outcome while not being significantly associated with any change in laboratory parameters (Fig. 4b, Additional file 1: Table S1, p: 0.033).

## Discussion

COVID-19 is associated with a high disease burden with nearly 20% of confirmed cases progressing towards critical illness [18]. The mortality rate in people infected with Sars-Cov-2 increases steeply with age, and fatal outcomes are almost exclusively seen in people older than 50 years [19]. Among the latter, the elderly in long-term care facilities (LTCFs) have been reported as high vulnerable to infections and at high risk for mortality. NFurthermore, patients with severe illness are likely to suffer substantial sequelae associated with a new physical disability, new cognitive impairment, and increased vulnerability to recurrent infection [14, 20, 21].

Currently, many drugs have been adopted worldwide for the treatment of Sars-Cov-2 pneumonia [22–27]; however, most of these drugs failed to demonstrate a clear clinical benefit. It is therefore of paramount importance to properly identify patients who are likely to develop critical illness for a timely pharmaco-invasive management while optimizing the use of limited resources.

In this study, we retrospectively evaluated and integrated laboratory parameters/variables of a homogeneous cohort of 45 elderly subjects from a long-term care facility with COVID-19, to identify potential prognostic factors able to better stratify patients’ clinical course and outcome. Radiographic and laboratory abnormalities, such as lymphopenia and elevated lactate dehydrogenase, are known to be common, but on their own are not specific for the diagnosis or the prognosis [1]. In line with previous findings, we observed that low lymphocytes/monocytes count as well as the high level of D-dimer in the peripheral blood significantly define a group of patients with a shattering outcome (practically 100% mortality rate). The integration of these three laboratory abnormalities well describes the known course and relative pathophysiology of COVID-19 [1, 10–13]. Indeed, profound lymphopenia occurs in individuals with COVID-19 when SARS-CoV-2 infects and kills T lymphocyte cells. The viral inflammatory response, consisting of both the innate and the adaptive immune response, impairs lymphopoiesis, and increases lymphocyte apoptosis. In the progression of the viral infection, when viral replication accelerates, epithelial-endothelial barrier integrity is compromised, exacerbating the inflammatory response. Finally, in severe COVID-19, fulminant activation of coagulation and consumption of clotting factors occur [28, 29].

Importantly, our results show that the systemic iron metabolism associated parameters, i.e. iron status, ferritin, and IFR, can help to better predict unfavorable outcomes and to monitor illness progression/regression in COVID-19 patients. Ferritin is a key mediator of immune dysregulation, especially under extreme hyperferritinemia, via direct immune-suppressive and pro-inflammatory effects, contributing to the cytokine storm. Previous laboratory findings highlighted that a consistent percentage of patients with elevated serum ferritin levels faced a higher probability to experience serious complications from COVID-19 [30, 31]. Accordingly, ferroptosis, a novel programmed cell death, has been suggested as an important cause of multiple organ involvement in COVID-19 and it might serve as a new treatment target [32–36].

In this study, we suggest a putative predictive value of the iron metabolism status as a marker of inflammation which potentially offers a new parameter to better stratify hospitalized patients—indeed, the increase of ferritin accompanied by low iron values, which result in elevated IFR, significantly segregates recovered and dead patients and correlates with a better prognosis in COVID-19 patients belonging to the so-called intermediate-risk group (4 deaths over 21 patients). Our hypothesis is that in this group, which represents the largest group, the early evaluation of IFR may function as a surrogate marker able to highlight the presence of a “smoldering” systemic inflammation, which could have been hidden by other confounding factors. In agreement, we observed a direct correlation between IFR and IL-6 only in the intermediate risk group. Furthermore, we found that the kinetic changes of ferritin levels, but not those of D-dimer and IL-6, correlate with COVID-19 progression. Indeed, we observed that almost exclusively in the recovering patients, the ferritin concentration peak at the time of hospital admission is followed by a gradual decrease during the second and the third week of the disease course. Accordingly, patients belonging to the high-risk group presented a significantly longer time to ferritin decrease.

Given the relevance of the COVID-19 pandemic and the lack of definitive early prognostic markers, we believe that any contribution which advances this field is highly valuable. Indeed, one of the major problems to evaluate the clinical status of COVID-19 patients at admission is the still simplistic stratification into mild, moderate and severe forms according to respiratory sign/symptoms and need for oxygen therapy and/or mechanical ventilation. The results of the present study suggest that the proposed laboratory findings could better and preventively stratify the patients in advance to the development of the clinical manifestations with a poor prognosis. It would be of interest to re-evaluate patients included in published clinical trials according to the suggested laboratory patterns of described here in order to assess whether these patterns better predict mortality of COVID-19 patients with mild, moderate and severe clinical presentation and whether these laboratory parameters better predict response to recommended therapies such as dexamethasone, remdesivir and heparin and monoclonal antibodies as well [37, 38].

This study has several limitations. First, the main weakness of the present study is the small sample size, which makes the reported findings hypothesis-generating. Second, this study included elderly patients from a unique long-term care facility, which represents an isolated outbreak in a rather homogenous population at risk that may not be representative of the overall heterogeneous general COVID-19 population. Testing a consecutive group of random COVID-19 patients, both retroactively and prospectively, will surely help to assess whether the conclusions reached from such a ‘population isolate’ will have general predictive value. Yet, it worth noting here that this study may have helped in generating robust evidence avoiding the confounding effects of multiple co-morbidities as in the general population.

## Conclusions

The main findings of this study generate the hypothesis that a combination of few laboratory parameters, and in particular iron status, D-dimer and lymphocyte count can better and preventively stratify COVID-19 patients with a poor prognosis in advance to the development of the clinical manifestations. Despite the inherent value of the present data for this population, its wide applicability to all COVID-19 patients requires validations in larger study cohorts, such as in patients included in clinical trials. This would allow to assess whether the proposed laboratory patterns better predict mortality of COVID-19 patients with mild, moderate and severe clinical presentation as well as the response to recommended therapies.

## Supplementary Information

**Additional file 1: Figure S1.** Boxplot, correlation plots, density plots and histograms reporting the association between each laboratory variable and Sex. The p values for overall and group-based correlation is reported in the top-right part of each image. **Figure S2.** Boxplot, correlation plots, density plots and histograms reporting the association between each laboratory variable and Age, respectively. The p values for overall and group-based correlation is reported in the top-right part of each image. **Figure S3.** Boxplot, correlation plots, density plots and histograms reporting the association between each laboratory variable and risk group, respectively. The p values for overall and group-based correlation is reported in the top-right part of each image. **Figure S4.** Boxplot, correlation plots, density plots and histograms reporting the association between each laboratory variable and survival, respectively. The p values for overall and group-based correlation is reported in the top-right part of each image.

## Data Availability

All data utilized, generated or analyzed during these studies are included in this published article.

## Abbreviations

SARS-CoV-2: Severe acute respiratory syndrome coronavirus 2 (COVID-19)
COVID-19: Coronavirus Disease-19
IFR: Iron-to-ferritin-ratio
IL-1: Interleukin-1
IL-6: Interleukin-6
qRT-PCR: Quantitative Real-Time-Polymerase Chain Reaction
BAL: Bronchoalveolar lavage
WBC: White blood count
NLR: Neutrophil-to-lymphocyte ratio
MLR: Monocyte-to-lymphocyte ratio
PLR: Platelet-to-lymphocyte ratio
HGB: Hemoglobin
PCA: Principal Component Analysis
Type 2 DM: Type 2 Diabetes Mellitus
COPD: Chronic Obstructive Pulmonary Disease
CKD: Chronic kidney disease
LTCF: Long-term care facilities
