# Supplementary material for: Combined lymphocyte/monocyte count, D-dimer and iron status predict COVID-19 course and outcome in a long-term care facility
Source: J Transl Med. 2021 Feb 17;19:79. doi: 10.1186/s12967-021-02744-2 (PMC7887565; doi:10.1186/s12967-021-02744-2)
Supplement: Supplementary file 1 — Additional file 1: Figure S1. Boxplot, correlation plots, density plots and histograms reporting the association between each laboratory variable and Sex. The p values for overall and group-based correlation is reported in the top-right part of each image. Figure S2. Boxplot, correlation plots, density plots and histograms reporting the association between each laboratory variable and Age, respectively. The p values for overall and group-based correlation is reported in the top-right part of each image. Figure S3. Boxplot, correlation plots, density plots and histograms reporting the association between each laboratory variable and risk group, respectively. The p values for overall and group-based correlation is reported in the top-right part of each image. Figure S4. Boxplot, correlation plots, density plots and histograms reporting the association between each laboratory variable and survival, respectively. The p values for overall and group-based correlation is reported in the top-right part of each image. [file 12967_2021_2744_MOESM1_ESM.docx]

**Combined lymphocyte/monocyte count, D-dimer and iron status predict COVID-19 course and outcome in a long-term care facility**

Flavia Biamonte^1,#,^ Cirino Botta^2,#^, Maria Mazzitelli^3^, Salvatore Rotundo^1^, Enrico Maria Trecarichi^3^, Daniela Foti^4^, Carlo Torti^3^, Giuseppe Viglietto^1^, Daniele Torella^1^, Francesco Costanzo^5^

**ADDITIONAL FILE 1**

**Additional file 1: Figure S1**

**
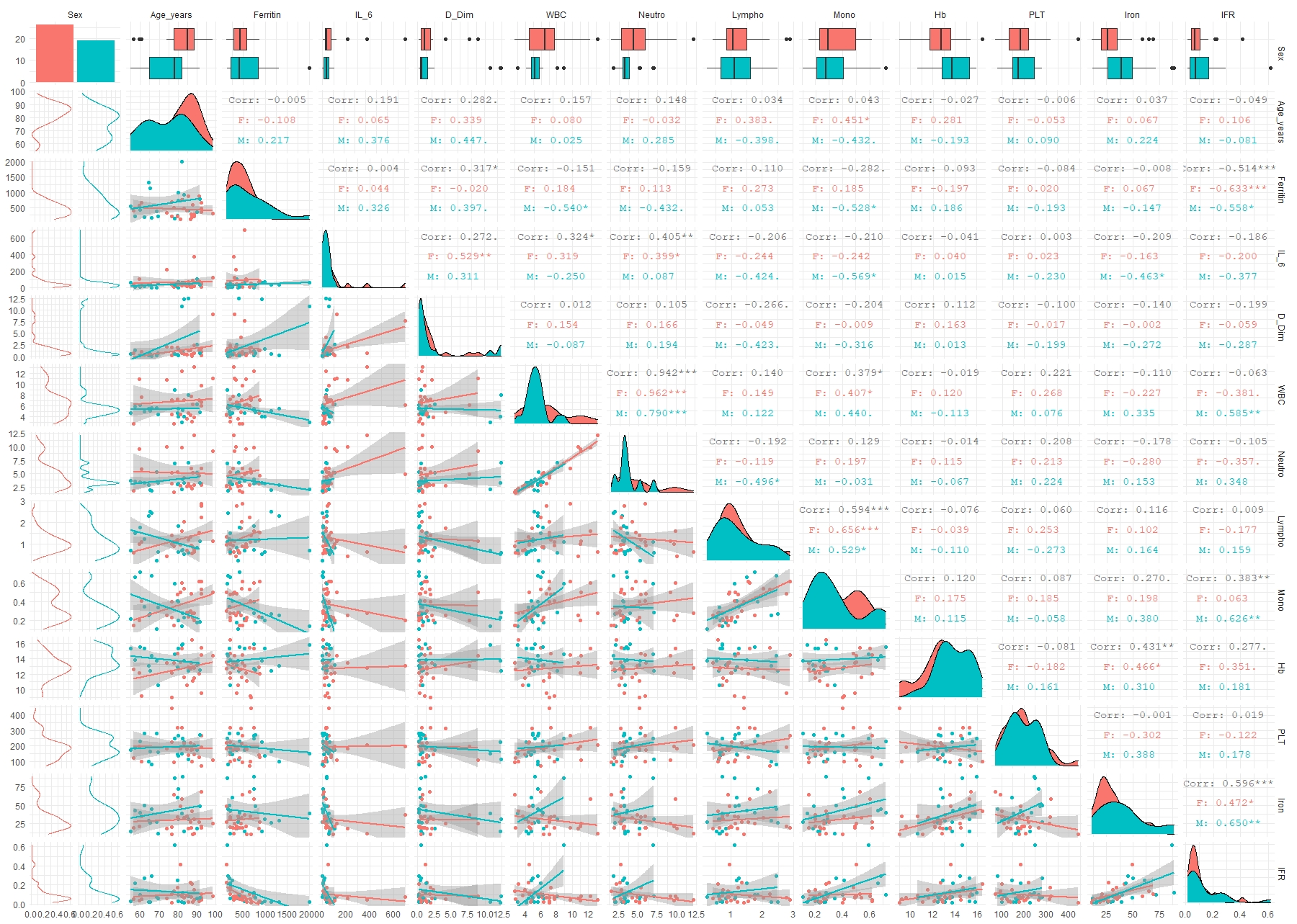
**

**Fig. S1:** Boxplot, correlation plots, density plots and histograms reporting the association between each laboratory variable and Sex. The p values for overall and group-based correlation is reported in the top-right part of each image.

**Additional file 1: Figure S2**


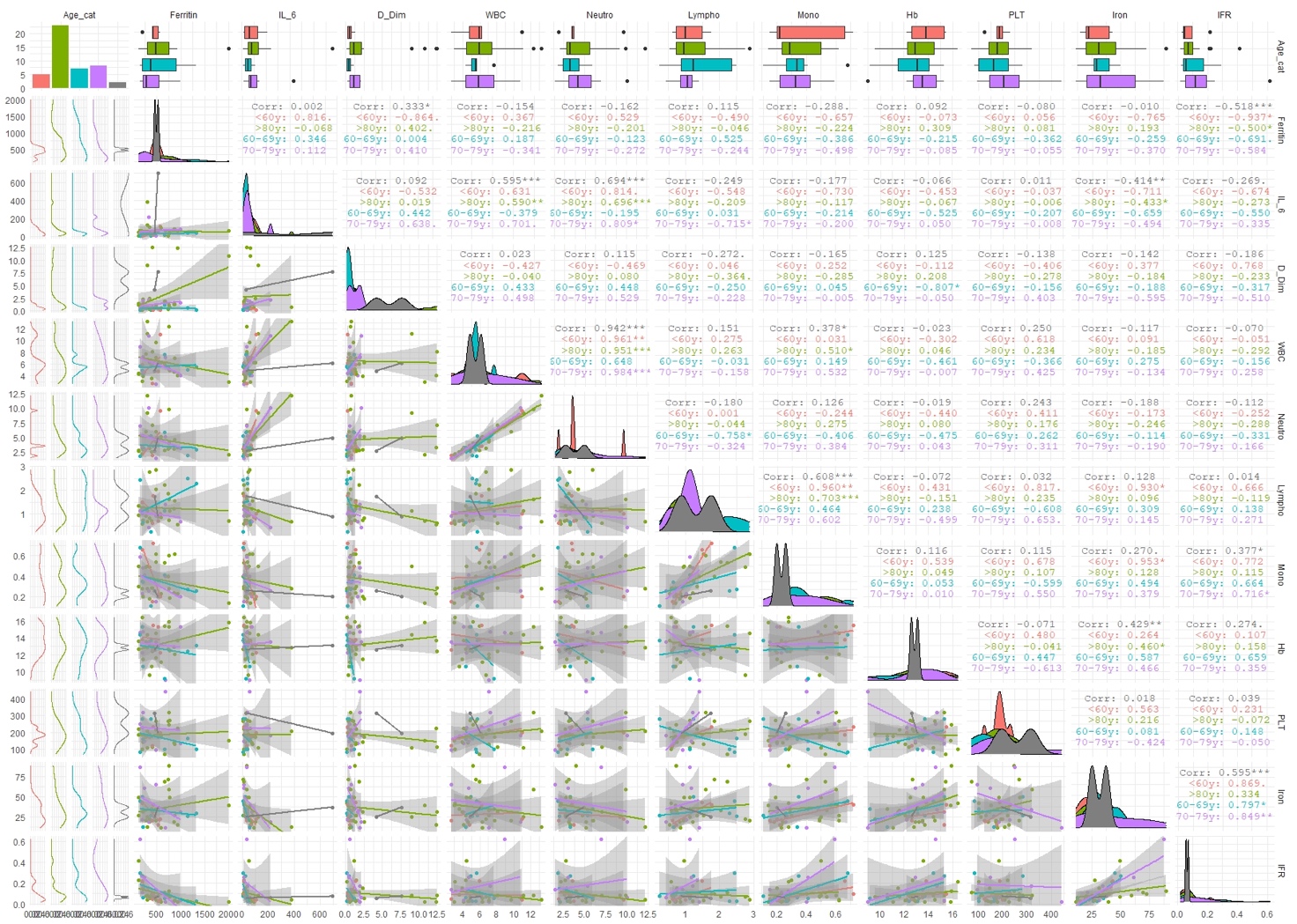


**Fig. S2:** Boxplot, correlation plots, density plots and histograms reporting the association between each laboratory variable and Age, respectively. The p values for overall and group-based correlation is reported in the top-right part of each image.

**Additional file 1: Figure S3**


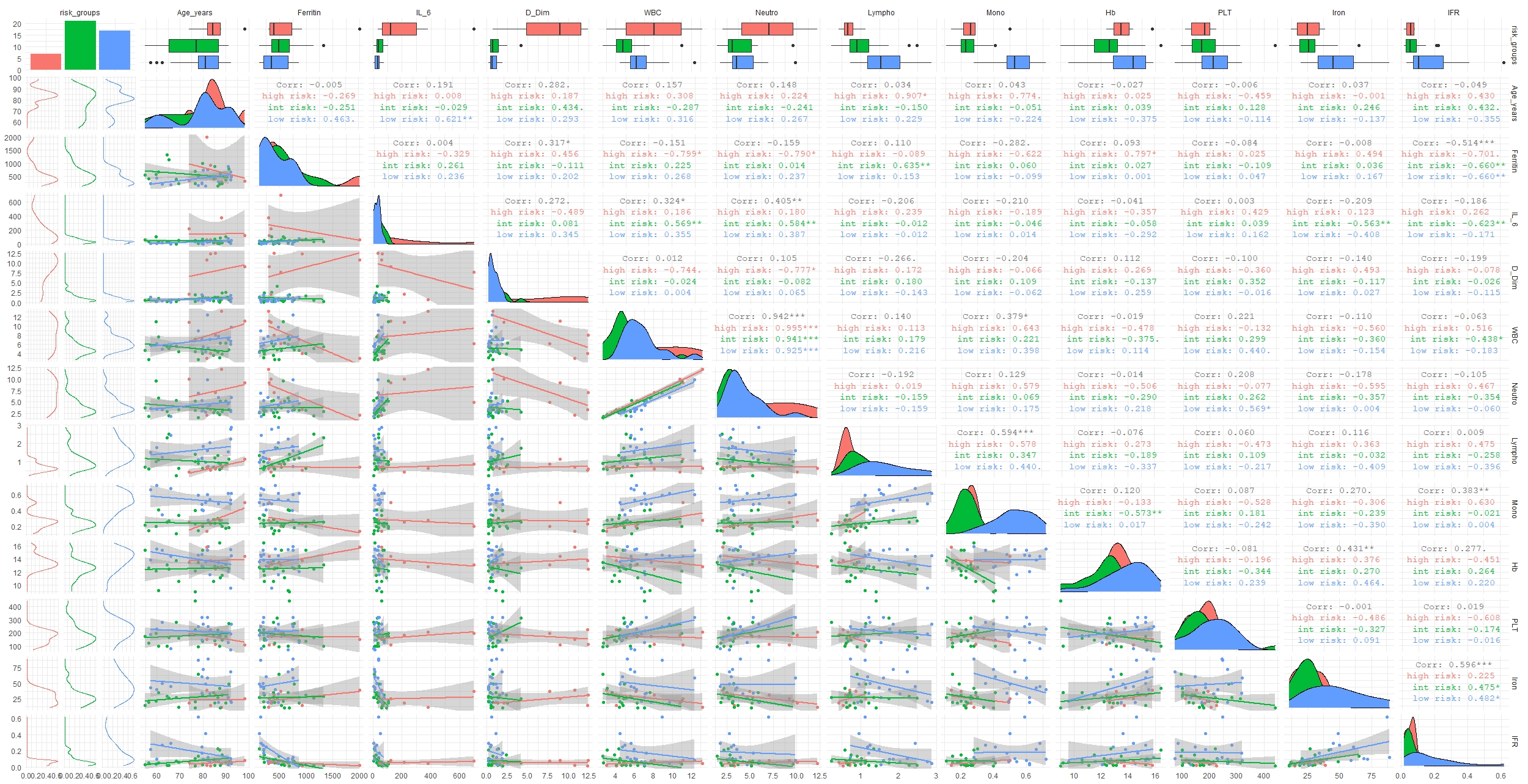


**Fig. S3:** Boxplot, correlation plots, density plots and histograms reporting the association between each laboratory variable and risk group, respectively. The p values for overall and group-based correlation is reported in the top-right part of each image.

**Additional file 1: Figure S4**


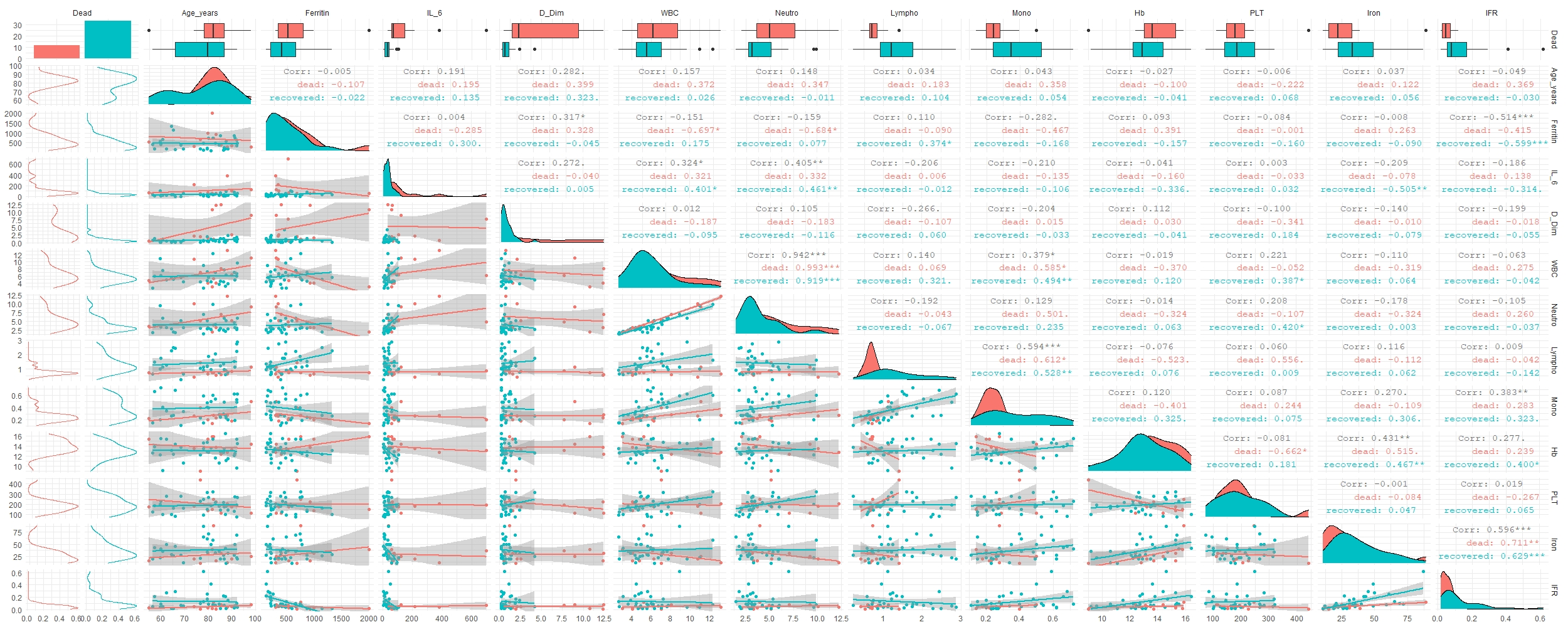


**Fig. S4:** Boxplot, correlation plots, density plots and histograms reporting the association between each laboratory variable and survival, respectively. The p values for overall and group-based correlation is reported in the top-right part of each image.

**Additional file 1: Table S1.**

| Comorbidity | p value  (Pearson Chi-square) |
| --- | --- |
| Malignancy | **0.047** |
| Psychiatric disease | 0.239 |
| Neurological disorder | 0.559 |
| Hypertension | **0.033** |
| Diabetes | 0.736 |
| COPD | 0.292 |
| CKD | 0.964 |
